# Supplementary material for: Using the Radial Distribution Function to Analyze Atomic Force Microscopy Images of Colloidal Systems
Source: Int J Mol Sci. 2024 Dec 30;26(1):210. doi: 10.3390/ijms26010210 (PMC11719932; doi:10.3390/ijms26010210)

Supplementary Materials to the Article

Using the radial distribution function to analyse AFM images of colloidal systems

*Sergey V. Kraevsky *, Anastasia A. Valueva, Maria O. Ershova, Ivan D. Shumov, Irina A. Ivanova, Sergey L. Kanashenko, Ilya A. Ryazantsev, Yuri D. Ivanov, Tatyana O. Pleshakova*

S1 Characterization of gold particles by electron microscopy (em)

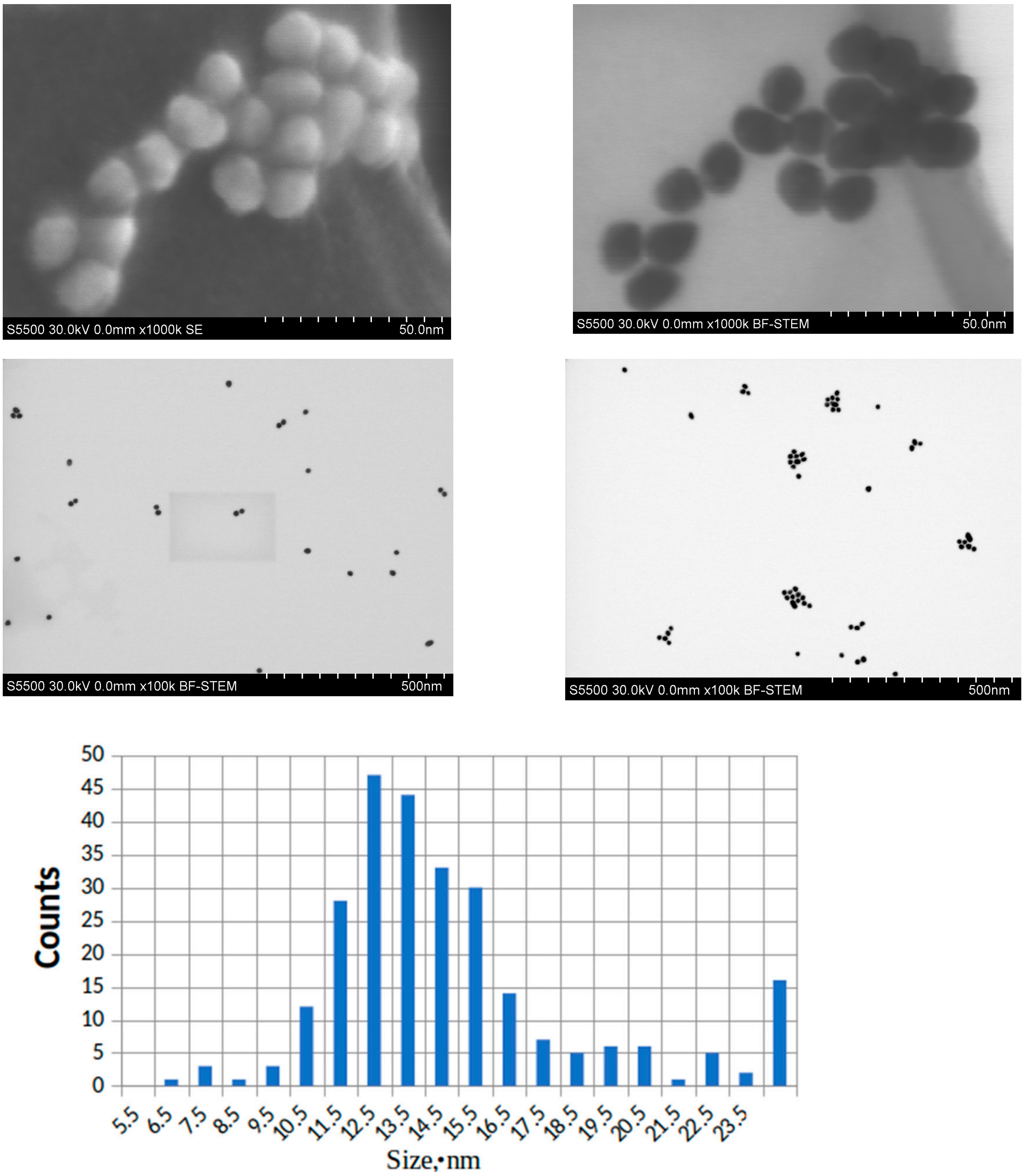

Figure S1: EM images of gold particles on the surface of carbon film and histogram of gold particle size distribution according to EM data. The EM study showed that the gold particles have sizes of 10–15 nm and are located on the carbon film both singly and in the form of "colonies".

S2 Constructing the RDF

In order to build the PDF, it is necessary to obtain a list of coordinates of the investigated objects on the surface. This, for example, can be done in Gwyddion software in several steps using the example of the original AFM image of gold particles: remove polynomial background; select grains using the Otsu method; export the x and y positions of the grains centers.

The Python script for RDF calculation is available at https://github.com/digital-ribosome/RDF .

S3 Gallery of AFM images of HRP sorbed on the surface

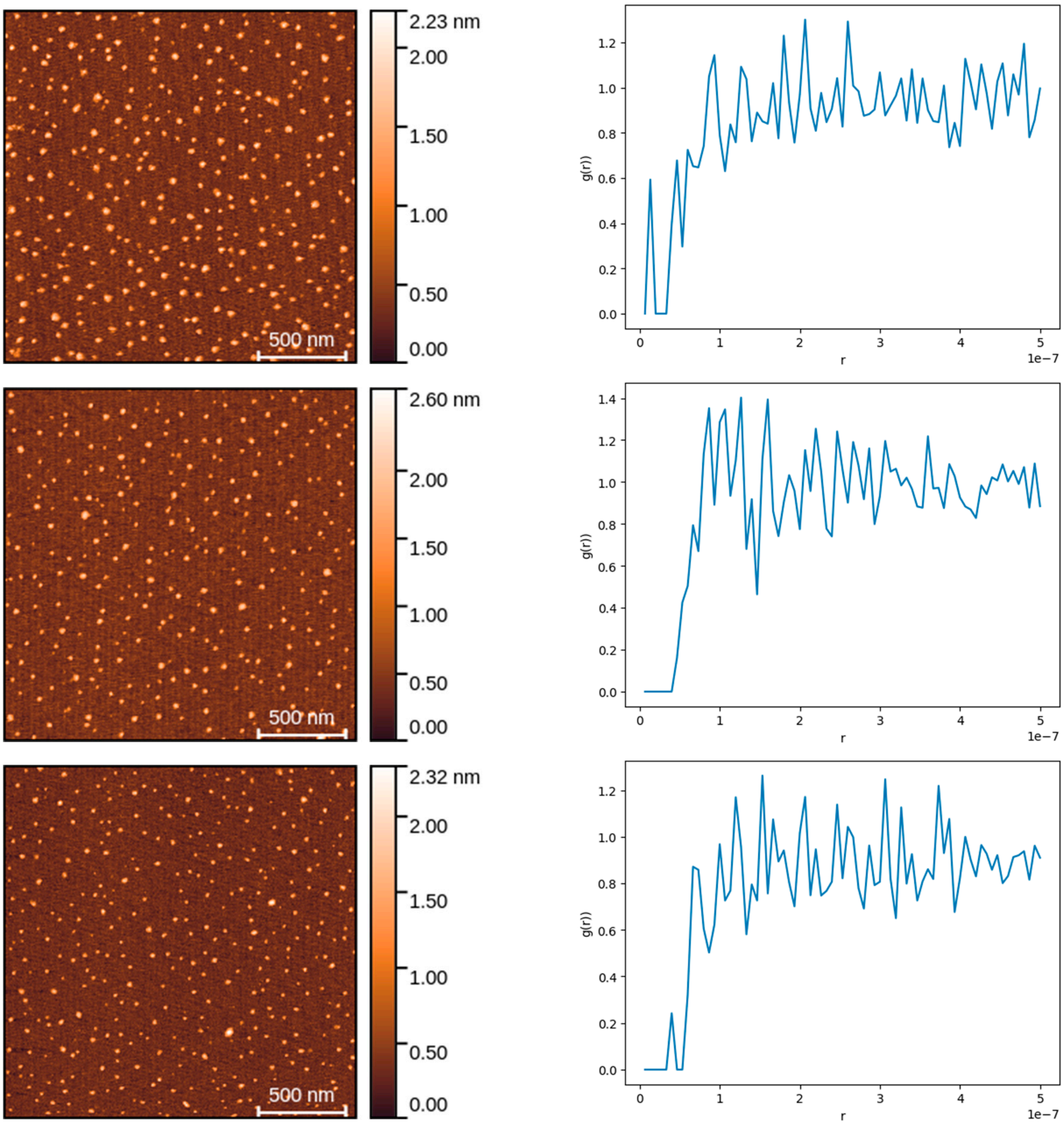

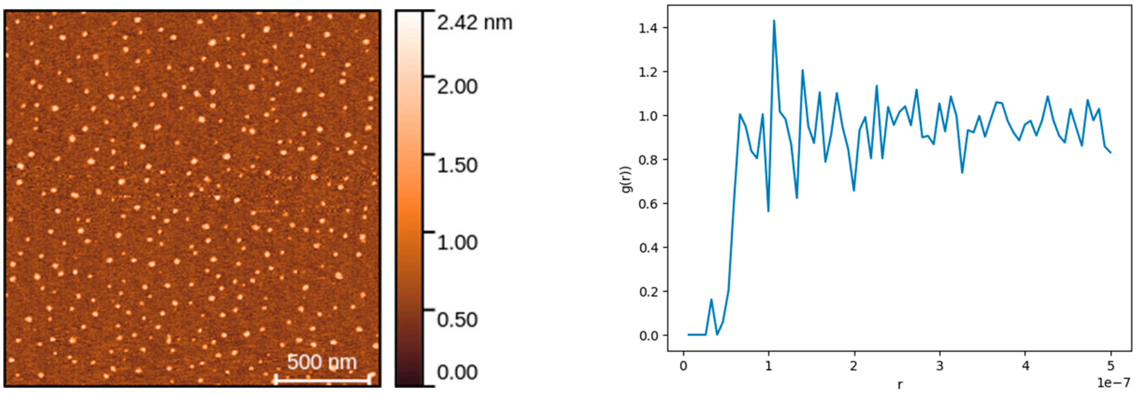

Figure S2: AFM images of the surface of freshly pierced mica (left panel) after incubation in 10-7 M HRP solution for 10 minutes and the radial distribution functions of the HRP corresponding to these images (right panel). Examples of images without aggregated structures.

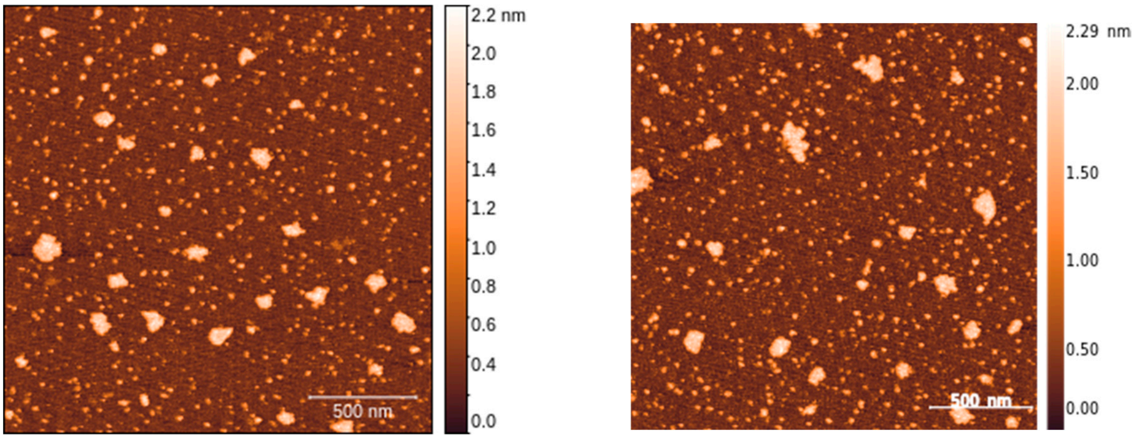

S4 Gallery of AFM images of AuNPs sorbed on the surface

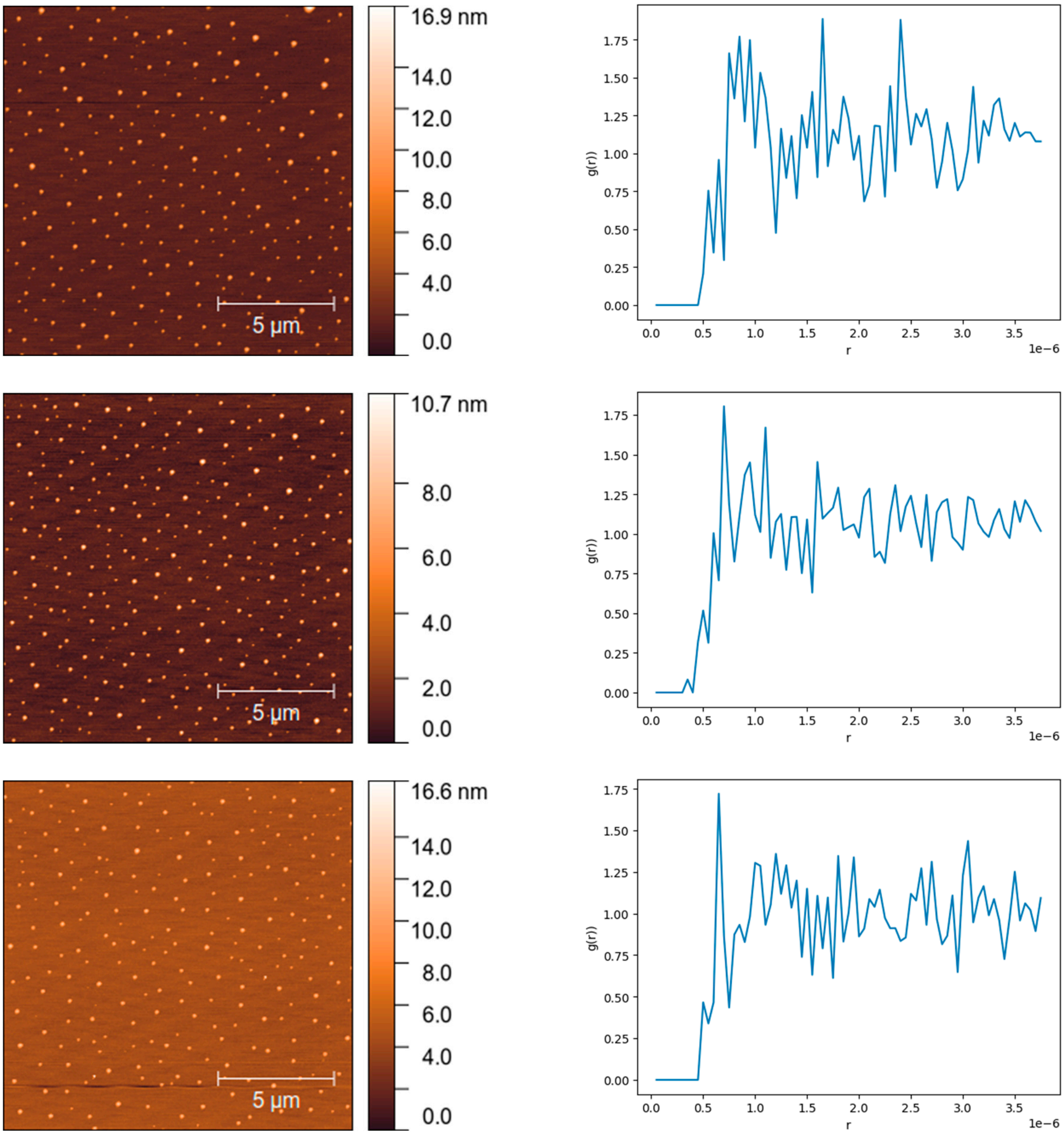

Figure S3: AFM images of the surface of freshly broken mica (left panel) after incubation on the surface of AuNP solution and drying at room temperature, and the radial distribution functions of the RDF corresponding to these images (right panel). Examples of images without aggregated structures.

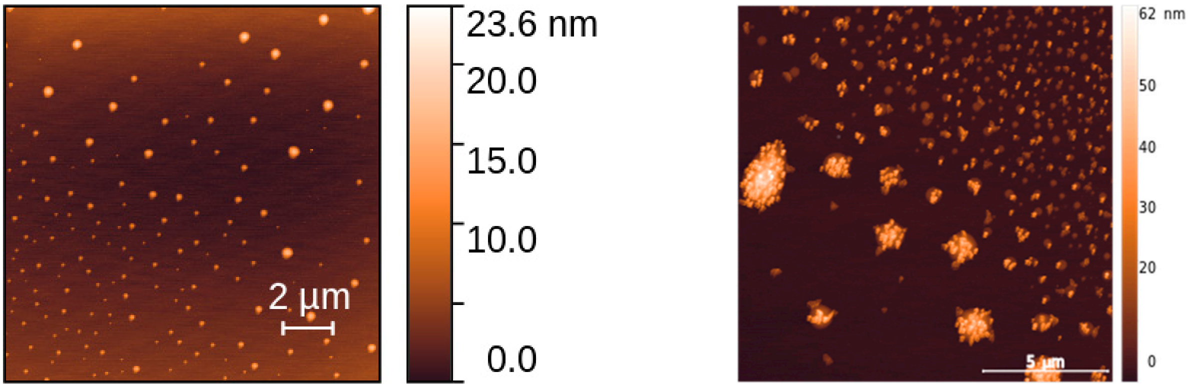

Supplement: Supplementary file 1 [file ijms-26-00210-s001.zip › ijms-3370346-supplementary.pdf]
